# Evaluation of the Immunity Activity of Glycyrrhizin in AR Mice

**DOI:** 10.3390/molecules17010716

**Published:** 2012-01-12

**Authors:** Xiao-Lan Li, Ai-Guo Zhou

**Affiliations:** 1 Ear-Nose-Throat (ENT) Department of the 1st Hospital Associated to the ShiHeZi University, ShiHeZi City 832008, XinJiang, China; 2 Acute Surgery Department of the 1st Hospital Associated to the ShiHeZi University, ShiHeZi City 832008, XinJiang, China; Email: zhouagsu207@yahoo.com.cn

**Keywords:** glycyrrhizin, immunity function, allergic rhinitis, TNF-α

## Abstract

In this study, we evaluated effect of glycyrrhizin on immunity function in allergic rhinitis (AR) mice. The AR mice model were induced by dripping ovalbumin in physiological saline (2 mg mL^−1^, 10 μL) into the bilateral nasal cavities using a micropipette. After the AR model was induced, mice were randomly divided into six groups: the normal control, model, lycopene 20 mg kg^−1^ (as positive control drug) group, and glycyrrhizin 10, 20, 30 mg kg^−1^ groups. After the sensitization day 14, lycopene (20 mg/kg BW) and glycyrrhizin (10, 20 and 30 mg/kg BW) were given orally for 20 days once a day. Mice in the normal control and model groups were given saline orally once a day for 20 days. Results showed that glycyrrhizin treatment could dose-dependently significantly reduce blood immunoglobulin E (IgE), interleukin-4 (IL-4), interleukin-5 (IL-5), interleukin-6 (IL-6), nitrous oxide (NO), tumor necrosis factor-alpha (TNF-α) levels and nitrous oxide synthase (NOS) activity and enhance blood immunoglobulin A (IgA), immunoglobulin G (IgG), immunoglobulin M (IgM), interleukin-2 (IL-2) and interleukin-12 (IL-12) levels in AR mice. Furthermore, glycyrrhizin treatment could dose-dependently significantly enhance acetylcholinesterase (AchE) activity and reduce substance P (SP) level in peripheral blood and nasal mucosa of AR mice. We conclude that glycyrrhizin can improve immunity function in AR mice, suggesting a potential drug for the prevention and therapy of AR.

## 1. Introduction

In Chinese traditional medicine, licorice (Gan Cao) remains one of the oldest and most commonly prescribed herbs, used in the treatment of numerous ailments ranging from tuberculosis to peptic ulcers [1]. Licorice has held claim for therapeutic use for fevers, liver ailments, dyspepsia, gastric ulcers, sore throats, asthma, bronchitis, Addison’s disease and rheumatoid arthritis and has been used as a laxative, antitussive and expectorant [2,3,4]. Among its most consistent uses are as a demulcent for the digestive system, to treat coughs, to soothe sore throats, and as a flavoring agent. Glycyrrhizin is a triterpene glycoside extracted from licorice root (*Glycyrrhiza glabra*). It consists of an 18-β-H-oleanane-type structure that combines with two sugar molecules (glucuronic acids) at the C-3 position. Glycyrrhizin and some analogues have various pharmacological effects such as anti-inflammatory, antitumorigenic and antihepatotoxic activities [5,6,7,8,9].

Allergic rhinitis (AR) is a very common and increasingly prevalent disease with high social and health care costs and has a significant impact on quality of life (QOL) [10,11,12]. AR is not life-threatening and is considered by many to be a trivial disease with mild symptoms. However, sufferers tell a different story. Assessment of the disease through both general health questionnaires and disease specific questionnaires shows a dramatic impairment of the QOL and usual daily activities with the patients with more severe disease experiencing the highest level of impairment [13]. Although the presence of the symptoms is perceived as a disturbing element in itself, in a recent patient survey, 85% of the patients felt that their daily activities, particularly those relating to their professional, personal and social life, their outdoor activities and their ability to function properly at work or at school and their sleep were impaired either moderately or severely. Furthermore, a large proportion of patients report that their disease causes sleep disturbances, such as trouble in going to sleep or awakening during the night and more than 50% report that they felt tired upon waking [14,15,16]. In this study, we selected glycyrrhizin and evaluated its effect on immunity function in AR mice.

## 2. Results

There were no significant differences (*P* > 0.05) in body weight between all groups at the beginning of the experiment (data not shown). After 20 days of medicine treatment, body weights in model control rats were markedly lower (*P* < 0.05) than those in normal control ones. No significant change was found in rats’ body weight after completion of the experiment in all medicine-treatment groups of rats. (*P* > 0.05). 

**Table 1:** Body weight in different groups.

Group	Body weight (g)
NC	37.12 ± 1.49
MC	34.15 ± 1.31 ^a^
lycopene 20 mg kg^−1^	34.97 ± 1.64
glycyrrhizin 10 mg kg^−1^	34.72 ± 1.73
glycyrrhizin 20 mg kg^−1^	35.01 ± 1.59
glycyrrhizin 30 mg kg^−1^	35.33 ± 1.82

^a^ *P* < 0.05, compared with NC group.

There were also no significant differences (*P* > 0.05) in body weight between medicine-treatment groups and MC group at the end of the experiment (Table 1). Compared with the NC group, blood IgE, IgA, and IgG levels in MC group rats were significantly (*P* < 0.01) increased, whereas blood IgA, and IgG levels were significantly decreased. Blood IgM levels in MC group rats were slightly (*P* > 0.05) decreased. The glycyrrhizin significantly (*P* < 0.01) reduced blood IgE, and enhanced IgA, and IgG levels in a concentration dependant manner up to a concentration of 30 mg/mL. The reference (positive control) lycopene also significantly decreased blood IgE, and enhanced IgA levels in rats. The glycyrrhizin showed stronger immunity-stimulating activity than the lycopene (Table 2).

**Table 2:** Effect of glycyrrhizin treatment on blood IgE, IgA, IgG and IgM levels.

Group	IgE (ng/mL)	IgA (g/L)	IgG (g/L)	IgM (g/L)
NC	20.39 ± 1.41	3.98 ± 0.23	16.32 ± 1.74	3.07 ± 0.21
MC	35.07 ± 1.43 ^b^	1.43 ± 0.12 ^b^	7.68 ± 0.54 ^b^	2.73 ± 0.17
lycopene 20 mg kg^−1^	29.78 ± 1.42 ^c^	2.07 ± 0.19 ^c^	15.77 ± 1.05	3.03 ± 0.24
glycyrrhizin 10 mg kg^−1^	30.28 ± 1.37 ^c^	1.64 ± 0.12	8.21 ± 0.78 ^c^	3.04 ± 0.19
glycyrrhizin 20 mg kg^−1^	24.27 ± 1.28 ^d^	2.61 ± 0.14 ^d^	10.29 ± 0.94 ^d^	3.01 ± 0.21
glycyrrhizin 30 mg kg^−1^	21.56 ± 2.15 ^d^	3.21 ± 0.16 ^d^	13.02 ± 1.14 ^d^	2.98 ± 0.23

^b^ *P* < 0.05, compared with NC group; ^c^ *P* < 0.05; ^d^ *P* < 0.01, compared with MC group.

Table 3 shows the results of blood IL-2, IL-4, IL-5, IL-6 and IL-12 levels in all groups of rat. Blood IL-4, IL-5 and IL-6 levels in MC group were significantly higher than those in NC group, whereas blood IL-2 and IL-12 levels in MC group were markedly lower. Blood IL-4, IL-5 and IL-6 levels in glycyrrhizin-treated rats were significantly decreased, whereas blood IL-2 and IL-12 levels in glycyrrhizin-treated rats were significantly increased when compared to MC group rats. Moreover, the effect was dose-dependent. The reference (positive control) lycopene also enhanced or decreased blood IL-2, IL-4, IL-5, IL-6 and IL-12 levels in AR rats, but the effect wasn’t significant.

**Table 3:** Effect of glycyrrhizin treatment on blood IL-2, IL-4, IL-5, IL-6 and IL-12 levels.

Group	IL-2 (pg/mg)	IL-4 (pg/mg)	IL-5 (pg/mg)	IL-6 (pg/mg)	IL-12 (pg/mg)
NC	16.42 ± 1.07	51.41 ± 1.11	3.05 ± 0.13	118.47 ± 8.69	37.29 ± 1.09
MC	9.32 ± 0.78 ^b^	58.07 ± 1.43 ^b^	5.21 ± 0.24 ^b^	153.03 ± 10.77 ^b^	28.69 ± 1.67 ^b^
lycopene 20 mg kg^−1^	10.54 ± 0.85	55.78 ± 1.42	4.84 ± 0.19	148.44 ± 12.31	30.21 ± 1.98
glycyrrhizin 10 mg kg^−1^	13.05 ± 0.97 ^d^	55.28 ± 1.37	4.27 ± 0.23 ^c^	137.57 ± 11.57 ^c^	33.87 ± 1.75 ^d^
glycyrrhizin 20 mg kg^−1^	15.11 ± 1.06 ^d^	53.27 ± 1.28 ^c^	3.76 ± 0.16 ^d^	128.42 ± 9.09 ^d^	35.21 ± 2.05 ^d^
glycyrrhizin 30 mg kg^−1^	15.98 ± 1.12 ^d^	51.56 ± 1.15 ^d^	3.28 ± 0.14 ^d^	120.62 ± 8.93 ^d^	37.08 ± 1.99 ^d^

^b^ *P* < 0.05, compared with NC group; ^c^ *P* < 0.05; ^d^ *P* < 0.01, compared with MC group.

Table 4 shows the results of blood NO, TNF-α levels and NOS activity in all groups of rat. Blood NO, TNF-α levels and NOS activity in MC group were significantly higher than those in NC group. Blood NO, TNF-α levels and NOS activity in glycyrrhizin-treated rats were significantly decreased. Moreover, the effect was dose-dependent. The reference (positive control) lycopene also significantly decreased blood NO, TNF-α levels and NOS activity in AR rats.

**Table 4:** Effect of glycyrrhizin treatment on blood NO, TNF-α levels and NOS activity.

Group	NO (μmol/L)	TNF-α (pg/mL)	NOS/(U/mL)
NC	63.82 ± 4.29	80.31 ± 5.94	21.65 ± 1.85
MC	159.43 ± 13.74 ^b^	163.29 ± 13.77 ^b^	59.42 ± 1.97 ^b^
lycopene 20 mg kg^−1^	123.57 ± 11.05 ^d^	130.14 ± 10.62 ^d^	45.12 ± 2.68 ^d^
glycyrrhizin 10 mg kg^−1^	131.54 ± 9.84 ^d^	134.28 ± 12.93 ^d^	41.63 ± 2.95 ^d^
glycyrrhizin 20 mg kg^−1^	92.37 ± 6.72 ^d^	102.41 ± 8.93 ^d^	33.07 ± 1.47 ^d^
glycyrrhizin 30 mg kg^−1^	72.66 ± 5.36 ^d^	89.37 ± 6.05 ^d^	26.87 ± 1.21 ^d^

^b^ *P* < 0.05, compared with NC group; ^d^ *P* < 0.01, compared with MC group.

In nasal mucosa AchE activity was reduced significantly (*P* < 0.01) in MC group rats as compared to normal control rats. Administration of glycyrrhizin for 20 days dose-dependently significantly enhanced nasal mucosa AchE activity in glycyrrhizin-treated group rats compared to MC group rats. The reference (positive control) lycopene also increased the activity of nasal mucosa AchE in AR rats, but result wasn’t significant (*P* > 0.05) (Table 5). 

**Table 5:** Effect of glycyrrhizin treatment on AchE activity in nasal mucosa.

Group	AchE activity (U/mg)
NC	0.3072 ± 0.0115
MC	0.2127 ± 0.0136 ^b^
lycopene 20 mg kg^−1^	0.2328 ± 0.0185
glycyrrhizin 10 mg kg^−1^	0.3347 ± 0.0317 ^d^
glycyrrhizin 20 mg kg^−1^	0.4203 ± 0.0298 ^d^
glycyrrhizin 30 mg kg^−1^	0.5032 ± 0.0243 ^d^

^b^ *P* < 0.05, compared with NC group; ^d^ *P* < 0.01, compared with MC group.

The level of Substance P (SP) in peripheral blood and nasal mucosa of MC group rats was significantly (*P* < 0.01) enhanced as compared to normal control rats. Oral administration of glycyrrhizin (10, 20 and 30 mg/kg body weight) for 20 days lowered the elevated values to nearly near normal (Table 6). The reference (positive control) lycopene also decreased the level of Substance P (SP) in peripheral blood and nasal mucosa in AR rats, but result wasn’t significant (*P* > 0.05).

**Table 6:** Effect of glycyrrhizin treatment on SP level in peripheral blood and nasal mucosa.

Group	Peripheral blood (pg/mL)	Nasal mucosa (pg/mL)
NC	85.38 ± 5.94	50.53 ± 6.03
MC	157.04 ± 13.29 ^b^	97.29 ± 10.67 ^b^
lycopene 20 mg kg^−1^	148.39 ± 13.05	91.32 ± 9.73
glycyrrhizin 10 mg kg^−1^	124.25 ± 13.11 ^d^	80.15 ± 9.05 ^c^
glycyrrhizin 20 mg kg^−1^	104.31 ± 9.49 ^d^	69.42 ± 7.77 ^d^
glycyrrhizin 30 mg kg^−1^	90.63 ± 7.37 ^d^	60.11 ± 7.14 ^d^

^b^ *P* < 0.05, compared with NC group; ^c^ *P* < 0.05, ^d^ *P* < 0.01, compared with MC group.

## 3. Discussion

Allergic rhinitis (AR) results from a complex allergen-driven mucosal inflammation occurring from a vicious cycle between resident and infiltrating inflammatory cells, a number of inflammatory mediators including cytokines and neurotransmitters leading to sensory nerve activation, plasma leakage, and congestion of venous sinusoids [17]. Because of its chronic nature, long term medications are required for therapy. Currently available therapeutic agents have major adverse effects, so it is imperative to find drugs, preferably non-steroidal in nature, which can be taken for a long time without any adverse effects.

With this basis, we selected a natural compound, glycyrrhizin, a major anti-inflammatory compound in licorice, to test its effects on OVA-induced allergic rhinitis in mice. Glycyrrhizin has been clinically used as an antiallergic agent and also as treatment for chronic hepatitis for more than 30 years [18]. Yuan *et al*. [19] recently reported the results of experiments using colitis induced by acetic acid installation, which is a model of acute colitis [20]. They found that glycyrrhizin reduced colonic injury with the suppression of nuclear factor-κB, TNF-α, and intercellular adhesion molecule-1 in the affected mucosa. So far, glycyrrhizin exhibits a number of pharmacological effects, including antiinflammation, anti-ulcer, anti-allergy, anti-carcinogenesis, and immunomodulation [21,22,23,24]. The compound is also used as a potential therapeutic agent for several virus diseases, including chronic hepatitis, acquired immunodeficiency syndrome, and herpes infection [25,26,27,28]. In our present study, body weight of AR mice was significantly lower than in normal control mice. We supposed that digestive function of AR mice was greatly weakened so as to affect food intake and as a result, body weight was greatly decreased. Many investigators have used total serum immunoglobulin E (IgE) and eosinophil count for evaluating allergic disease [29,30,31,32]. IL-4 is one of the key mediators for allergic response. It was first recognized as a B-cell growth factor and among other properties, it enhances antigen presenting capacity of these cells and is essential for the IgE synthesis [33,34,35]. IL-5 levels have been found to be significantly increased in symptomatic subjects with allergic diseases [36]. Positive correlations have also been shown between the levels of eosinophil mediators and IL-5 and the symptoms of seasonal AR [37]. Other reports have indicated that IL-5 acts directly as a chemoattractant and as an activator for eosinophil recruitment in allergic inflammatory disease [38,39]. In this report, we demonstrated that glycyrrhizin significantly reduces OVA-induced reduces OVA-specific IgE, IL-4, IL-5 and IL-6 levels in blood. On the other hand, it prevents the decrease in total IgA, IgG, IgM, IL-2 and IL-12 levels in serum in AR mice. These results indicate that glycyrrhizin modulates the general immunity in AR mice. In addition, glycyrrhizin does not affect the body weight in comparison to glycyrrhizin untreated mice. This result was in agreement with our previous work [40].

Nitric oxide (NO) plays a key role in the pathophysiology of airway diseases and the control of airway inflammation [41,42,43]. NO is formed by the enzyme NO synthase (NOS) from the semiessential amino acid L-arginine, and three isoforms of this enzyme have been identified in human airways [44,45]. The third isoform of NOS (iNOS) is inducible by proinflammatory cytokines and is found in several types of cells, including inflammatory cells and epithelial cells. There is increasing evidence that endogenous NO may increase airway inflammation. Recent evidence suggests that it may be derived from the very high levels in the paranasal sinuses. However immunohistochemical studies suggest that NO may also be derived from NOS expressed in the nasal mucosa. This may help to support our previous study, in which nasal NO was increased in patients with allergic rhinitis and reduced after treatment with nasal glucocorticosteroids [46]. In the present study, glycyrrhizin significantly reduced blood NO, TNF-α levels and NOS activity.

Both histamine and substance P are well known as itch-producing substances that can induce sneezing and nasal rubbing when applied locally [47,48]. In addition, it is considered that nasal instillation of capsaicin elicits sneezing mediated by releasing substance P from the sensory nerve [49,50]. In the field of neuroscience, substance P (SP) is a neuropeptide: an undecapeptide that functions as a neurotransmitter and as a neuromodulator [51,52]. It belongs to the tachykinin neuropeptide family. Substance P and its closely related neuropeptide neurokinin A (NKA) are produced from a polyprotein precursor after differential splicing of the preprotachykinin A gene. 

AchE activity is frequently used as a biomarker to evaluate toxic effects of herbicides and pesticides. The activity of this enzyme is extremely important for many physiological functions, such as locomotion, prey location, predator evasion and orientation toward food. Muscular AchE represents the largest pool of cholinesterases in the body, and is important for controlling the muscular function [53,54]. Inhibition of the AChE enzyme results in a build up of acetylcholine causing a continuous and excessive stimulation of the nerve/muscle fibres which leads to tetany, paralysis and eventual death. Measurement of AChE inhibition in aquatic organisms has already been used as a biomarker of effects of neurotoxic contaminants [55,56,57,58,59,60].

The results of the present study show that in control individuals the activity of AchE was higher in muscle than in brain. On the contrary, glycyrrhizin significantly enhanced AchE activity and inhibited SP level in peripheral blood and nasal mucosa in a dose dependent manner in AR mice.

## 4. Experimental

### 4.1. Preparation of Glycyrrhizin

Glycyrrhizin was extracted from the dried roots of licorice according to our previous method [40]. The obtained sample was analyzed with HPLC and the purity of the resultant glycyrrhizin was 93%.

### 4.2. Animals

Male, Kunming mice (27–30 g, body weight, BW), 2 month-old, were kept in polyacrylic cages (38 cm × 23 cm × 10 cm) with ten animals per cage. They were fed a standard rat pellet diet and water ad-libitum and maintained under standard laboratory condition (temperature 24–26 °C, relative humidity 50–60%).

### 4.3. Allergic Rhinitis Model

Mice were sensitized by subcutaneous injection (sc) of antigen ovalbumin (1 mg) mixed with aluminum hydroxide adjuvant (80 mg) in saline (1 mL) in the footpad, neck, back and groin on the first day. Local challenge was performed every day from day 20 to day 26 by dripping ovalbumin in physiological saline (2 mg mL^−1^, 10 μL) into the bilateral nasal cavities using a micropipette. Mice in normal control were dripped saline. All procedures were in accordance with China’s National Animal Care Guidelines.

### 4.4. Animal Grouping and Treatment

Mice were randomly divided into six groups: the normal control, model, lycopene 20 mg kg^−1^ (as positive control drug) group, and glycyrrhizin 10, 20, 30 mg kg^−1^ groups. After the sensitization day 14, lycopene (20 mg/kg BW) and glycyrrhizin (10, 20 and 30 mg/kg BW) were given orally once a day for 20 days. Mice in the normal control and model groups were given saline orally for 20 days once a day.

### 4.5. Measurement of Blood IgE, IgA, IgG, IgM, IL-4, IL-2, IL-5, IL-6, IL-10, TNF-α Levels

Blood IgE, IgA, IgG, IgM, IL-4, IL-2, IL-5, IL-6, IL-10, TNF-α levels were measured using commercially available enzyme-linked immunosorbent assay (ELISA) kits (Sigma). SP levels in nasal mucosa were determined using enzyme linked immunosorbent assay.

### 4.6. Measurement of Serum Nitric Oxide Level

The blood was centrifuged at 500 rpm for 5 min, then equal volumes of serum, acidic Griess reagent (pH 2) and glycine buffer (40 μL, 100 μM of glycine, 100 μM of NaCl and 40 μM of HCl) was added and then incubated for 15 min at 37 °C. The normal group sample was incubated with saline whereas control and treated group samples were incubated with compound 48/80 (1 μg/mL). The absorbance was measured at 546 nm [61,62].

### 4.7. Determination of Nitric Oxide Synthase Activity

The activity of nitric oxide synthase was determined by the estimation of nitrite concentration in the serum sample using the Greiss reaction assay [63]. The measurement of nitrate/nitrite concentration or of total nitrate and nitrite concentration (NOx) is routinely used as an index of NO production. In this method, nitrite was first treated with a diazotizing reagent, *i.e.*, sulfanilamide (SA), in acidic media to form a transient diazonium salt. This intermediate was then allowed to react with a coupling reagent, *N*-naphthylethylenediamine (NED), to form a stable azo compound.

### 4.8. Determination of AChE Activity

AChE activity was measured using acetylcholine bromide as the enzyme substrate [64]. AChBr and hydroxylamine were from Sigma Co., and all other chemicals were of high quality and purchased from commercial suppliers. Nasal mucosa was homogenized in 0.1 M phosphate buffer (1 mL, pH 7.00) by using a small chilled glass Teflon tissue grinder. Homogenates were centrifuged at 10,000 r.p.m. for 15 min. at 5 °C in a refrigerated centrifuge (GS-6r, Beckman, Mountain View, CA, USA). The deposits were discarded and the supernatant used for enzyme activity determination which carried out in 3–4 replicates, and the optical densities were measured against blank at 540 nm, using a spectrophotometer (Spectronic 1201, Milton Roy Co., Rochester, NY, USA). The results were calculated by constructing a standard curve.

### 4.9. Statistical Analysis

The data for various biochemical parameters were analysed using ANOVA and the group means were compared by Duncan’s multiple range test. Values were considered statistically significant when *P* < 0.05.

## 5. Conclusions

In conclusion, glycyrrhizin can enhance immunity function in AR mice.
